# Chemotherapy of acute myeloid leukaemia in adults: Medical Research Council.

**DOI:** 10.1038/bjc.1979.9

**Published:** 1979-01

**Authors:** 

## Abstract

Two hundred and fifty patients with acute myeloid leukaemia (AML) were randomized between 2 regimens of chemotherapy: TRAP and BARTS III. Overall, patients randomized to TRAP, which was the more intensive of the 2 regimens, fared slightly better (P = 0.06) than those on BARTS III. However, the improvement in survival associated with more intensive chemotherapy was substantial only for patients who had favourable prognostic features at presentation, such as a normal total leucocyte count, or absence of palpable liver, or, especially, age under 40. Indeed, for patients under 40, those allocated to the more intensive regimen (TRAP) lived considerably longer than those allocated to BARTS III (P less than 0.002) while for patients over 40 there was no material difference in survival between patients on the 2 protocols. It thus appears that intensive chemotherapy is likely to be more effective when favourable prognostic features are recorded.


					
Br. J. Cancer (1979) 39, 69

CHEMOTHERAPY OF ACUTE MYELOID LEUKAEMIA IN ADULTS

MEDICAL RESEARCH COUNCIL

Report prepared by H. Cuckle, D. A. G. Galton and R. Peto for the Medical Research Council's

lWorking Party on Leukaemia in Adults on the Sixth Trial (Chemotherapy).

The mneinmbers of the Working Party ov-er the period of the trial w erie SIR .JOHN DACIE (Chairinan),
D. A. G. GALTON (Secretary), K. D. BAGSHAWE, P. BARKHAN, A. J. BELLINGHAMT,
E. K. BLACKBURN, S. CALLENDER, I. W. DELAMORE, SIR RICHARD DOLL,
J. DURRANT, J. J. FENNELLY, I. D. FRASER, F. J. G. HAYHOE, J. R. HOBBS, J. INNES,
H. E. M. KAY, G. W. MARSH, G. A. McDONALD, I. C. M. MAcLENNAN, M. G. NELSON,
R. PETO, R. POWLES, 0. S. ROATH, B. E. ROBERTS, J. STUART, R. B. THOMPSON,

G. WETHERLEY-MEIN, J. A. WHITTAKER and E. WILTSHAW.

Receive(d 2 August 1978 Acceptedl 16 October 1978

Summary.-Two hundred and fifty patients with acute myeloid leukaemia (AML)
were randomized between 2 regimens of chemotherapy: TRAP and BARTS III.
Overall, patients randomized to TRAP, which was the more intensive of the 2
regimens, fared slightly better (P=0'06) than those on BARTS III. However, the
improvement in survival associated with more intensive chemotherapy was sub-
stantial only for patients who had favourable prognostic features at presentation,
such as a normal total leucocyte count, or absence of palpable liver, or, especially,
age under 40. Indeed, for patients under 40, those allocated to the more intensive
regimen (TRAP) lived considerably longer than those allocated to BARTS III
(P<0-002) while for patients over 40 there was no material difference in survival
between patients on the 2 protocols. It thus appears that intensive chemotherapy
is likely to be more effective when favourable prognostic features are recorded.

IN THE Medical Research Council's 5th
acute myeloid leukaemia (AML) trial, a
4-drug treatment schedule, RAMP (dauno-
rubicin (Rubidomycin), cytosine arabino-
side, mercaptopurine, prednisone) used in
the previous trial was compared with one
or other of two 2-drug schedules in which
cytosine arabinoside (AraC) was adminis-
tered in combination with either thio-
guanine or daunorubicin (DR). The
AraC/DR 2-drug combination, which
seemed to be the best of these 3 treatments,
was not statistically significantly better
than the other 2 (MRC, 1974; 1975). How-
ever, this treatment is unlikely to be
much worse than, and was not more toxic
than, the other 2. Consequently, we
decided to carry over this "best arm",
or something very like it (i.e. "BARTS

III" chemotherapy, see below), from the
5th trial into the 6th trial, part of which
we now report.

The Medical Research Council's "6th
AML trial" in fact consists of 2 entirely
separate trials. At some centres the
"AML 6 immunotherapy" trial was or-
ganized to determine the value of adding
immunotherapy to a standard form,
(BARTS III) of chemotherapy closely
resembling the best arm of the 5th trial
(MRC, 1978; Harris et al., 1978). At other
centres the "AML 6 chemotherapy" trial
was organized to determine whether more
intensive chemotherapy would be more
effective than BARTS III. In 1972 a pilot
trial in the MRC Leukaemia Unit of a
more intensive multiple-drug regimen
(TRAP) had yielded promising results

Reprinits from Dr H. Cuckle, Departmenit of the Regius Professor of Medicine, Radcliffe Infirmary, Oxford.

MEDICAL RESEARCH COUNCIL

(Spiers et al., 1977). The MRC 6th AML
chemotherapy trial which we now report,
therefore compared, by random allocation
of newly diagnosed AML patients, these 2
regimens, TRAP and BARTS III, to see
whether the extra toxicity expected from
TRAP would be compensated for by
higher remission rates and better survival.

THE PROTOCOLS

BARTS III Chemotherapy

Remis8ion-induction  therapy.-Five-day
courses of daunorubicin (DR) and cytosine
arabinoside (Ara-C) with 5-day intervals
between the last day of one course and the
first day of the next.

Each 5-day course consisted of daily i.v.
injections of Ara-C 70 mg/M2 (2 mg/kg) plus,
on the first day, DR 55 mg/M2 (1.5 mg/kg)
by fast injection just before the first injection
of Ara-C. (In the MRC 5th trial schedule, the
Ara-C was administered in 12-hourly doses,
but the schedules were otherwise identical.)

Courses were to be continued until marrow
aspirated on the day before the next course
fell due showed hypoplasia with few or no
residual blasts, after which 2 or 3 weeks
without treatment were allowed. When
marrow regeneration was recorded, and
upward trends in the haemoglobin concentra-
tion and neutrophil and platelet counts were
apparent, one further (consolidation) course
was to be administered before proceeding to
the maintenance schedule. If blast cells per-
sisted in the marrow when the 5th course was
due, the doses of DR and Ara-C were to be
increased by 20%.

Maintenance chemotherapy.-Five-day
courses of (i) Ara-C and thioguanine (TG),
and of (ii) Ara-C with DR on Day 1 only,
were alternated at monthly intervals until
the onset of relapse. The doses of Ara-C
and DR were the same as for induction
therapy, and the daily dose of TG was
70 mg/M2 (2 mg/kg) by mouth. Maintenance
chemotherapy was started 1 month after the
last consolidation course.

The TRAP Programme

The TRAP programme was designed in the
MRC Leukaemia Unit by Dr A. S. D. Spiers.
The remission-induction schedule was based
on one of the schedules included in the 5th
trial, but with thioguanine substituted for

mercaptopurine, and with courses repeated at
14-day intervals. The cyclical maintenance
therapy was designed to expose the residual
leukaemia cells to several different cytoxic
drugs, in the hope of reducing the rate of
emergence of drug-resistant lines and thus
deferring the onset of relapse.

TRAP remission-induction therapy.-Six 5-
day courses of TRAP were administered in
12 weeks, with a 9-day interval between the
last day of one course and the first day of the
next.

Each course consisted of TG, 100 mg/M2
daily for 5 days by mouth; DR, 40 mg/M2
i.v. on the first day only; Ara-C, 100 mg/M2
i.v. or i.m. daily for 3 days in the 1st course,
4 days in the 2nd course and 5 days there-
after; plus prednisolone, 30 mg/M2 daily for
5 days by mouth. After the 3rd course, the
doses of DR and Ara-C were to be increased
by 20% on successive courses, if the blood-
count trends permitted, to a maximum of
100 mg of DR and 300 mg of Ara-C.

TRAP cyclic maintenance therapy.-Each
cycle of 20 weeks consisted of 2 courses of
COAP (4 weeks), 3 of TRAP (6 weeks), 2 of
POMP (4 weeks), and 3 of TRAP (6 weeks).

COAP was a 5-day course followed by a
9-day interval: cyclophosphamide, 100 mg/M2
daily by mouth for 3 days in the first course,
increasing, if possible, to 5 days; predniso-
lone, 100 mg b.d. for 5 days; vincristine 2 mg
i.v. on the first day only; and Ara-C, 100
mg/M2 i.v. or i.m. daily for 5 days.

POMP was a 5-day course followed by a
9-day interval: prednisolone, 100 mg b.d. for
5 days; vincristine, 2 mg i.v. on the first day
only; methotrexate, 7-5 mg/M2 i.v. or i.m.
daily for 3 days; and, for the first of each
pair of courses, mercaptopurine, 300 mg/M2
for 3 days. Each course could be delayed for
1 week if the blood counts were considered
suboptimal.

Eligibility for entry to the trial

All AML patients, irrespective of age,
were eligible for entry to the trial, provided
they had not received any prior treatment
with cytotoxic drugs. Patients suffering from
undifferentiated blast-cell leukaemia were
entered into this trial if aged over 25, ex-
cluded from it if aged under 20, and admitted
or not at the clinician's discretion if aged
between 20 and 25. Patients with leukaemia
supervening on pre-existing haematological

70

CHEMOTHERAPY OF AML

disorders were not eligible for entry to the
present study, nor were patients presenting
at the 4 centres which were undertaking the
MRC immunotherapy trial (MRC, 1978;
Harris et al., 1978). Patients who were
entered were immediately randomized, in
equal proportions, between TRAP and
BARTS III.

STATISTICAL METHODS

These are as described in the report on
statistical methods to the Medical Re-
search Council's Leukaemia Steering Com-
mittee (Peto et al., 1976; 1977). They
chiefly involve the plotting of Kaplan-
Meier life-table estimates of the percent-
ages alive at various times up to 21 years
after entry, after achieving remission, or
after relapse, to illustrate various patterns
of survival, and the calculation of logrank
P-values to test the statistical significance
of any apparent differences in survival or
remission duration. For the latter, exact
variance calculations were performed
(ibid., Statistical Note 7) and continuity
corrections were not used (ibid., p. 38).
(Because follow-up is complete to 3 years
after entry, and to 2 2 years after remission
induction for all long survivors, these life-
table plots of survival from entry or from
remission reduce to simple graphs of the
observed percentages alive at various
times.)

PATIENTS ENTERED

Between January 1973 and November
1974, 250 patients newly diagnosed as
suffering from AML were admitted to the
trial. 125 were allocated randomly to
receive BARTS III chemotherapy, and
125 to the TRAP programme of treatment.
Intake was ended when an interim analysis
of the duration of survival did not, in the
autumn of 1974, indicate any substantial
difference between the 2 treatments,
for some participants feared that side

effects and complications associated with
the TRAP programme would be unaccept-
able, unless the results of treatment were
considerably better than those obtained
with the BARTS III schedule. (Prolonged
follow-up has shown, however, that there
is some survival advantage in using TRAP,
and that its toxicity is not severe.)

DATA COLLECTED

At presentation most of the following
information was recorded for each of 244
of the 250 patients: platelet and haemo-
globin levels; leucocyte (WBC), blast-cell,
neutrophil and monocyte counts; whether
or not there were gum deposits, skin
deposits and haemorrhagic manifestations;
and whether or not the liver, spleen and
nodes were palpable.

Patients were followed up until 1
January 1978, and the dates of any re-
missions, relapses or deaths were recorded*
and the causes of death were sought. Each
patient has thus been followed to death
or for at least 3 years.

RESULTS

The statistical analysis of the results is
presented in 9 tables in the Appendix
(AT I to AT IX) and in 5 figures showing
the duration of survival in different sub-
groups of patients.

Relation between treatment and survival
(AT I)

Patients randomized to TRAP did
slightly better than those on BARTS III
(Fig. 1). Remission was achieved by 82
patients (32% of those randomized), 42 of
whom were on TRAP and 40 on BARTS
III. Whilst the remission rate was similar
for both treatments, there was a small,
but almost statistically significant (P=
0-06), difference between their survival

* The date of death was not known for one patient who had refused treatment and was believed to have
died. Her date of death was taken to be 60 days after randomization. The date of remission was not known for
one patient who was still alive one year after randomization, and it was assumed that she achieved remission
after 60 days.

7 1

72                       MEDICAL RESEARCH COUNCIL

curves. Patients randomized to TRAP    the duration of survival after relapse was
had a 30% probability of surviving for  statistically significantly better for TRAP
one year compared with 23% for those on  than for BARTS III (X2 - 6X1, DF = 1,
BARTS III. Whilst the duration of first  0X02 > P > 0X01). This produced a slight-
remissions was similar for both treatments, ly better prognosis after remission for

I_n

. v

0. 8
0

.-I

c 3:   0. 6

; _

E - 0

0

0 0
.!: E ?
= o
._

ffi    0. 2

o.o

L,.,

.          L

L ~--',_

,             .TRA.P

LI1

Time from entry to trial (years)

(a)

1 n

.. V

0. 8

-C

C,
._ 0

c_     0.6

E 0

_ _

:. to  0. 4
= E
_ o

X-     0. 2

o. o

--- II.---

AL ,

BARTS 111          Li ---

X      -,..  .... ... .

s ~~~~~~~~~~~~~~~~~~~~~~~~~~~~~~~~~~~~~~~~~~I

D                          1                         2

Time from onset of remission (years)

(b)

FIG. 1.-(a) Duration of survival for 125 patients randomized to BARTS III and 125 randomized to

TRAP.

(b) Duration of survival after first remission for the 40 patients on BARTS III and the 42 on TRAP
who achieved remission.

(c) (opposite) Estimated duration of survival after first relapse for the 31 patients on BARTS III
and the 31 on TRAP who relapsed in their first remission.

CHEMOTHERAPY OF AML

TRAP

Time from first relapse (years)

FIG. 1 (C)

AGE 0-39

Time from entry to trial (years)

FIG. 2.-Duration of survival for the 74 patients aged <40, the 92 aged 40-59 and the 84 aged > 60.

patients on TRAP (P = 0-13) who had a
43% probability of surviving 2 years after
remission, compared with 20% for those
on BARTS III.

The improvement effected by using a
more intensive programme (TRAP) rather
than a gentle one (BARTS III) was more
marked in the patients with more favour-

able prognostic features. This was so
whether the patients were sub-divided
into "favourable" or "unfavourable" prog-
nosis groups on the basis of age, WBC,
blast-cell count, haemoglobin level or on
whether the liver was palpable at presen-
tation. In each case the more intensive
(TRAP) schedule appeared to be no better

1. 0

0. 8
0. 6
0. 4
0. 2

C

:._

c

a-

c

E

._

o
0

0. 0

1. 0
0. 8

0.4
c :3

._     0. 6

0 0
E =

qZ _

a)-
O a,

vL  0. 4
_~ E

= O

-

X-     0. 2

o. o

73

MEDICAL RESEARCH COUNCIL

L, ~ ~ ~ ~ ~   _

I ^ r  1

2

Time from entry to trial (years)

(a)

TRAP

Time from entry to trial ( years)

(b)

FIG. 3.-(a) Duration of survival for the 39 patients on BARTS III and the 35 on TRAP, who were

aged <40.

(b) Duration of survival for the 86 patients on BARTS III and the 90 on TRAP who were aged > 40.
(c) (opposite) Duration of survival after first remission for the 15 patients on BARTS Ifl and the
20 on TRAP, who were aged <40 and achieved remission.

(d) Duration of survival after first remission for the 25 patients on BARTS III and the 22 on TRAP,
who were aged > 40 and achieved remission.

74

1.0
0. 8

.3

ac

c i   0.6

0

1 o

C, -

>..   0.4
-E
=-

CL    0. 2

0. 0

1.0

-C

C -a

E =
cs

_; o

E .

= E2

_= E
- _
=

0. 8
0.6
0.4
0.2
0. 0

X - s

I

1

CHEMOTHERAPY OF AML

L-I

TRAP

I                   ------------l
BARTS II1 I

2

Time from onset of remission (years)

FIG. 3(c)

AL

II--

+~~~~~~~~~~~  I

BARTS  III  L1  ,

,   --

II _ _

1                                2
Time from onset of remission (years)

FIG. 3(d)

than the gentler (BARTS III) programme
for bad prognosis patients, but among
patients whose prognosis was better than
average the more intensive antileukaemic
treatment appeared to be somewhat more
effective. Appendix Tables (AT) II-VII
show this in detail.

Relation between age at presentation and
survival (AT II)

The age of patients ranged from 2 years
old to 77, with 74 patients (30%) under
40. The proportion who achieved remission
declined considerably with increasing age
and the survival curves showed a strong

1. 0

0. 8

0.6

75

, l

C._=

cn I

c. I

E O9
e- a)

= o
._ 0
Z _
m

0.4

0. 2

0. 0

1. 0

a _

E-I

. O:
M _

.- =

- O

*- 2.
8

o-"

=CE

0. 8
0. 6
0. 4
0. 2
0. 0

0

I                                                                                                        I
I                                                                                                     .1

MEDICAL RESEARCH COUNCIL

1. 0

.-I

c 3:
.; _

E -
w o5

o; z

- E
_- o

D -i
co

0. 8
0. 6
0. 4
0. 2

0. 0

WBC 5-9 x 109/I

Time from entry to trial (years)

FiG. 4.-Duration of survival for the 28 patients with WBC 5-9 x 109/1, the 81 with WBC 10-49 x 109/1

and the 62 with WBC > 50 x 109/1. The 73 with WBC < 5 x 109/1 (not shown) had similar survival
to those with 10-49.

trend with age (P<0-0001, Fig. 2). How-
ever, there was no significant trend with
age in survival from the onset of remission,
though 40% (6/15) of patients aged 60 or
more relapsed within 6 months of remis-
sion, compared with 16% (11/67) of the
younger patients.

Among patients under 40 years old,
those on TRAP had the higher remission
rate (57%  as against 38%  on BARTS
III), and, moreover, patients aged under
40 who achieved remission also survived
longer thereafter if they were on TRAP
than if they were on BARTS III (P= 0.04).
Overall survival, which includes the sur-
vival before remission is achieved, the
duration of remission, and the length of
survival after relapse was, therefore,
highly significantly better on TRAP than
on BARTS III for patients under 40
(P 0-002). However, among patients of
40 and older there was no material dif-
ference between the 2 treatments in
remission rate or in survival following re-
mission and so in overall survival (Fig. 3)
although survival after relapse appeared
to be better on TRAP.

Relation between WBC at presentation and
survival (AT III)

This is shown in Fig. 4. (Patients with
WBC<5 x 109/1 had similar survival to
those with 10-49 x 109/1 and are not
represented in the figure.) There was a
significant (P=0 02) tendency for those
with higher WBC to fare worse, and 38%
of patients whose WBC was less than
10 x 109/1 achieved remission compared
with 30% for those with 10 x 109/1 or
more. There was a small but not statistic-
ally significant effect of original WBC on
the prognosis after remission. Thus, al-
though the WBC is statistically signifi-
cantly associated with survival, the
strength of the association is much less
than in the case of acute lymphoblastic
leukaemia, where the presenting WBC is
of critical importance both for overall
survival and for length of first remission.

Among patients with WBC < 10 x 109/1,
those randomized to TRAP had a 40 %
remission rate compared with 35 %  for
BARTS III, and a better prognosis after
remission than those on BARTS III,
though this was not statistically signi-

76

I

CHEMOTHERAPY OF AML

ficant (P = 0 * 1 6). Hence, among these low-
WBC patients, overall survival was better
for those treated with TRAP (P-0 06).
There were, however, no material dif-
ferences between the treatments among
patients with a WBC of 10 x 109/1 or more.

The combined effects of aye and WBC on
survival (AT IV)

For the 29 patients with WBC less than
10 x 109/1 and who were also under 40,
those on TRAP fared highly significantly
better than those on BARTS III (P

0 007), although since we are describing
the single selected subgroup which best
illustrates our point, this nominal P-value
is exaggerated. Among those with WBC
of 10 x 109/1 or more and who were also
under 40, there was also some deficit of
deaths for those on TRAP though this
was not statistically significant. Among
patients aged over 40 there was no dif-
ference between treatments, whether the
WBC was high or low. This suggests that
age alone (over or under 40) may be a
sufficient determinant of whether inten-
sive or moderate chemotherapy is prefer-
able, but that perhaps for patients aged

around 40 or so a WBC below 1X0 109/1
indicates more intensive treatment.

Relation of blast-cell count at presentation
and survival (AT V)

Patients with <5 x 109/1 of blast cells
had a remission rate of 39%, compared
with 27% for those with 5 x 109/1 or more,
and there was a statistically significant
downward trend (P=0-005) in the survival
curves with increasing blast-cell counts.
This trend was not reduced when allowance
was made for age. There was a small but
not statistically significant effect on prog-
nosis after remission.

It has been shown that patients with
blast cells > 100 x 109/1 have a poor prog-
nosis (Harris, 1978) and this was confirmed
in the present study. The 21 such patients
in this group had significantly worse prog-
nosis (P=0.003), with only 11 surviving
the first month and 3 surviving more than
one year.

Among patients whose blast-cell counts
were <5 x 109/1, prognosis was somewhat
better with TRAP, while among patients
with blast-cell counts )5 x 109/1 the
difference between the treatments was
smaller.

Liver not palpable

2

Time from entry to trial lyearsl

FIG. 5. Duration of survival for the 78 patients for whom the liver was recorded as palpable and the

166 for whom the liver was recorded as'not palpable at presentation.

1. 0

_a
a

a 0
2-

10

o OL
= E

-
._

L-o

CL

0. 8
0.6
0.4
0. 2

0. 0

.                            l

77

I

MEDICAL RESEARCH COUNCIL

Relation between haemoglobin level at
presentation and survival (AT VI)

The 59 patients with haemoglobin levels
of IO g/100 ml or more had a 41 %  re-
mission rate compared with 310% for those
whose Hb was less than 10, and there was
a significant trend between the survival
curves, P 0-02. Among patients with
Hb levels >10 g/100 ml, 48 % on TRAP
achieved remission compared with 330o on
BARTS III. For those with lower levels
the rates were 29% and 3300 respectively.
Within any one particular Hb category
(<7.5, 7.5-9.9, 10+) the difference be-
tween TRAP and BARTS III was not
statistically significant, perhaps because
of small numbers, but an analysis of the
effects of treatment retrospectively strati-
fied for Hb (by summation of the observed
and expected numbers in the lower part
of AT VI) yields P-values like those in
Table I.

Relation between state of the liver at
presentation and survival (AT VII)

Physicians were asked to report whether
the liver was palpable at presentation.
Although this is an unreliable assessment,
patients reported to have had a palpable
liver (almost one-third of the total) had a
considerably worse prognosis than the
others (P=0.0003, Fig. 5). Their 6-month
survival rate was 3500 compared with
4800 among patients with no record of
liver enlargement, and the one-year rates
were 150% and 320% respectively. These
patients had a remission rate of only 26%
and there was also a statistically signi-
ficant (P 0.005) difference in survival
after remission. Among patients entering
remission, only 2000 of those originally
noted with a palpably enlarged liver
survived for 2 or more years, while 3900 of
those whose liver was not palpable did so.
The relevance of (recorded) liver enlarge-
ment was highly statistically significant
in the patients over 40 (P 0 003) and was
also present, though less markedly, in the
younger group. This suggests that the
presence of a palpable liver is an indepen-

dent prognostic feature. Among patients
who were reported not to have an enlarged
liver at presentation, those on TRAP had a
39%0  one-year survival rate compared
with 24% for those on BARTS III; the
difference between the survival curves was
statistically significant (P-00 1). This
treatment difference was even greater
among those who were also under 40
(X2   165, DF    1, P < 00001). In con-
trast, among patients who were reported
as having an enlarged liver, those ran-
domized to BARTS III actually fared
slightly better than those on TRAP,
though this difference was not statistically
significant (P  0.13).

Relation between other features recorded at
presentation and survival (AT VIII)

Several other presentation features were
related to prognosis, but the effects were
very small. Patients with platelet counts
>50 x 109/1 had a slightly higher chance of
remission, and tended to survive a little
longer. Similarly, the presence of an
enlarged spleen, gum or skin deposits, or
haemorrhagic manifestation at presenta-
tion were associated with a lower remission
rate.

Effects of retrospective stratification on
treatment (AT IX)

Finally, to check that the treatment
comparison was not biased by any chance
allocation of too many good-prognosis
patients to one treatment arm, the treat-
ment x2 was computed after retrospective
stratification for various indices of prog-
nosis. As may be seen, stratification did
not result in any striking changes in the
treatment effect, which remained at P

01l in all patients together. It is only in
the good prognosis subgroups (see above)
that any marked superiority of TRAP is
apparent.

DISCUSSION

The duration of survival in AML
depends largely on whether or not the
patient enters remission, arbitrarily de-

78

CHEMOTHERAPY OF AML

fined as a blast-cell count in the marrow
below 500 when regeneration has occurred
after remission-induction therapy. This
definition provides only a crude index of
the number of leukaemia cells surviving
remission-induction therapy, and cannot
distinguish cases in which the number of
surviving cells is small from those in which
it is much larger but still within the 50o
limit of the definition. If the rate of pro-
liferation of surviving leukaemia cells were
constant whatever their total number, the
time to detectable relapse, and therefore
the duration of remission, would be longest
when the number of surviving cells was
least. If the proportion of leukaemia cells
destroyed is related to the intensity of
therapy, more intensive therapy should
leave fewer surviving cells, and should
increase both the percentage of remissions
induced and the duration of remission.
The rate of proliferation of the surviving
leukaemia cells might also be lowered by
increasing the intensity of maintenance
therapy, while the risk of emergence of
drug-resistant cells might be reduced by
regularly changing the drug combinations
administered for maintenance therapy.
The TRAP programme was designed with
these aims, and the results of the uncon-
trolled pilot trial (Spiers et al., 1977) sug-
gested that the duration of remission had
been considerably prolonged over those in
published reports, the median duration
being 66 weeks (range 28-208 weeks). It
was not clear whether the increased per-
centage of remissions (60% of 25 patients)
would be repeatable in a larger series.

The present trial was designed to com-
pare the more intensive TRAP programme
with the less intensive BARTS III pro-
tocol in a multi-centre trial. By random
allocation 125 patients were treated ac-
cording to the TRAP programme and 125
according to the BARTS III protocol. It
is true that the overall results showed no
significant difference between the two
protocols in remission rate (320% for
BARTS III and 3400 for TRAP) or in
duration of survival (23% of BARTS III
patients and 300/ of TRAP alive at one

6

year) but there was an indication that for
the patients who entered remission, sur-
vival was superior in the TRAP group
(4300 alive 2 years after the onset of re-
mission compared with 3000 in the BARTS
III group). Patients with a blast-cell
count >100 x 109/1 at presentation have a
very poor prognosis (Harris, 1978) as do
those with hypergranular promyelocytic
leukaemia (MRC, 1975). When the 32
patients in the present study with either
of these features were removed from the
analysis, the duration of survival for
TRAP became significantly better than
for BARTS III (P-0.05).

When the patients were divided into
groups with and without favourable prog-
nostic features, TRAP proved to be
significantly better among the former in
respect of remission rate, overall survival
and survival after the onset of remission,
the last reflecting more the longer survival
after relapse than the longer duration of
remission.

If it is indeed true that the relative
merits of trial treatments are materially
different, or even opposite, for different
categories of patient, then quite large trials
will be needed in order to clarify this
reliably. Even in the present trials with
250 patients, there remains some doubt
about the extent to which artefacts of
chance have influenced the age-specific
differences between TRAP and BARTS
III, and in a trial with only 100 patients
these patterns might have gone unnoticed.

The most important prognostic feature,
as in previous trials (MRC, 1974; 1975)
was age (Fig. 2), the overall survival de-
creasing in progressively older age groups.
For the 74 patients under 40, the results
were markedly superior in the TRAP
group, for which, as compared with the
BARTS III group, there was 57 o and
38% of remissions, with 51% and 28% of
all patients surviving 2 years from the
onset of remission. Similar trends are seen
when the results are analysed according
to other independent features of prog-
nostic significance, including leucocyte
count at presentation (Table III), blast-

79

MEDICAL RESEARCH COUNCIL

cell count at presentation (Table V), Hb
concentration at presentation (Table VI)
and the presence or absence of palpable
liver enlargement at presentation (Table
VII). In each case the advantage of treat-
ment by the TRAP programme is chiefly
apparent in the groups with more favour-
able prognostic features, namely leuco-
cyte count < 10 x 109/1, blast-cell count
<5 X 109/1, Hb concentration >10 g/dl, or
liver not palpable.

For patients with more than one favour-
able prognostic feature at presentation,
e.g. age <40 and leucocyte count
<10 x 109/1, the apparent advantage of
TRAP was especially marked (Table IV),
but the numbers are small.

In contrast, for patients with unfavour-
able prognostic features, the TRAP pro-
gramme gave no advantage over the gentler
BARTS III protocol (Tables II-VII, Fig.
3b). The percentage of remissions was low
in both treatment groups because of
high fatality during remission-induction
therapy. This reflects the inability of poor-
risk patients to withstand the hazards of
prolonged marrow failure. Potentially
more effective therapy has no advantage
in these patients unless they can be kept
alive long enough to reap the benefits. It
is not immediately apparent why the
poor-risk TRAP patients who did enter
remission failed to show the same pro-
longation of survival as the good-risk
patients. A possible explanation was that
they tolerated the intensive maintenance
therapy poorly and so received inadequate
treatment. However, inspection of the
charts in the 2 groups does not show a
marked difference in the amount of treat-
ment received.

The present trial is the first in the series
of MRC trials of multi-drug therapy for
AML in which survival after complete
remission had been induced differed be-
tween treatments. In the groups of good-
risk patients concerned, the more intensive
treatment programme led to higher re-
mission rates and to longer survival after
relapse in those patients who entered
remission. For these patients, at least, the

inference must be that the future of
chemotherapy lies in more intensive treat-
ment rather than the gentler treatment
advocated by Burge et al. (1975). The
superior results of more intensive therapy,
administered with adequate supportive
care, have been shown in other trials and
form the basis of the current trial. Thus,
Stavem et al. (1977) using the TRAP pro-
gramme, reported 7/56 patients surviving
in complete remission for 4-6 years, having
been off therapy for 1-3 years. Even more
intensive remission-induction schedules
have been reported to give remission rates

80%     (Gale & Cline, 1977; Rees et al.,
1977) and the remissions reported in
elderly patients no doubt reflect the high
quality of supportive care. For the poor-
risk groups, it seems likely that improve-
ment in supportive care during remission-
induction therapy would reduce the risk of
early death, and so increase the chance of
entering remission. If this is true, more
intensive therapy might improve the
results of treatment in the same way as it
has in the good-risk groups, but while
supportive care is inadequate, more inten-
sive therapy offers no advantage, and its
use is difficult to justify because of the
extra toxicity, extra cost and the high
incidence of side effects.

The results of the present trial are not
quite as good for either TRAP or BARTS
III as in the original reports (Spiers et al.,
1977; Crowther et al., 1970). It is often
believed that multi-centre trials are in-
herently incapable of reproducing results
reported by those who carried out the
original trials, and that the standards of
practice at the participating centres are
somehow inferior to those at the originat-
ing centre. There is, however, a more likely
explanation. The results of a new form of
treatment administered to a group of
patients arise both from the intrinsic
merits of the treatment and from chance
factors which may operate to give rise to
results better or worse than the average
for that treatment. Even if the treatment
is, in fact, no more effective than conven-
tional treatment, superior results in the

so

CHEMOTHERAPY OF AML                        81

first series of patients treated will lead to
early publication, whereas indifferent or
inferior results will lead to the new
method being abandoned. A new treat-
ment that is, in fact, somewhat more
effective than conventional treatment is
certain to be reported with enthusiasm if
the early results are, by chance, strikingly
superior. Later trials involving larger
numbers of patients will confirm the
superiority of the treatment, but the
results are likely to be less striking than
those originally reported, as has been the
case in the present trial. It will be recalled
that the original figure of 62% of remis-
sions for the BARTS III protocol dropped
to 42 % in a subsequent report by the same
workers (Crowther et al., 1973).

We wish to thank the many colleagues who re-
ferred patients to the trial, Miss M. Gilham who
collected the data, Mrs P. Bide and Mrs R. Rohr-
basser who prepared the typescript. Thanks are also
due to Dr C. Costello who reviewed the charts of all
the patients who entered remission.

REFERENCES

BURGE, P. S., RICHARDS, J. D. M., THOMPSON, D. S.,

PRANKERD, T. A. J., SARE, M. & WRIGHT, P.
(1975) Quality and quantity of survival in acute
myeloid leukaemia. Lancet, ii, 621.

CROWTHER, D., POWLES, R. L., BATEMAN, C. J. T.

& 6 others (1973) Management of adult acute
myelogenous leukaemia, Br. Med. J., i, 131.

CROWTHER, D., BATEMAN, C. J. T., VARTEN, C. P.

& 4 others (1970) Combination chemotherapy
using L-asparaginase, daunorubicin, and cytosine
arabinoside in adults with acute myelogenous
leukaemia. Br. Med. J., iv, 513.

GALE, R. P. & CLINE, M. J. (1977) High remission

induction rate in acute myeloid leukaemia. Lancet,
i, 497.

HARRIs, A. L. (1978) Leukostasis associated with

blood transfusion in acute myeloid leukaemia. Br.
Med. J., i, 1169.

HARRIS, R., ZUHRIE, S. R., FREEMAN, C. B. &

6 others (1978) Active immunotherapy in acute
myelogenous leukaemia and the induction of
second and subsequent remissions. Br. J. Cancer,
37, 282.

MEDICAL RESEARCH COUNCIL (1974) Treatment of

acute myeloid leukaemia with daunorubicin,
cytosine arabinoside, mercaptopurine, L-aspara-
ginase, prednisone and thioguanine: results of
treatment with five multiple-drug schedules. Br.
J. Haematol., 27, 373.

MEDICAL RESEARCH COUNCIL (1975) The relation-

ship between morphology and other features of
acute myeloid leukaemia and their prognostic
significance. Br. J. Haematol., 31, (Suppl.), 165.

MEDICAL RESEARCH COUNCIL (1978) Immunotherapy

of acute myeloid leukaemia. Br. J. Cancer, 37, 1.
PETO, R., PIKT, M. C., ARMITAGE, P. & 7 others

(1976; 1977). Design and analysis of randomized
clinical trials requiring prolonged observation of
each patient. Br. J. Cancer, 34, 585 and 35, 1.

REES, J. K. H., SANDLER, R. M., CHALLENER, J. &

HAYHOE, F. G. J. (1977) Treatment of acute
myeloid leukaemia with a triple cytotoxic regime:
DAT. Br. J. Cancer, 36, 770.

SPIERS, A. S. D., GOLDMAN, J. M., CATOVSKY, D.,

COSTELLO, C., GALTON, D. A. G. & PITCHER, C. S.
(1977) Prolonged remission maintenance in acute
myeloid leukaemia. Br. Med. J., ii, 544.

STAVEM, P., GJEMDAL, T. & LY, B. (1977) Prolonged

remission maintenance in acute myeloid leukaemia.
Br. Med. J., ii, 831.

Notes added in proof

(1) By 1 December 1978 a further 2
patients had died. In a subsequent statis-
tical analysis the x2 for treatment became
2X99 for the total group, 9X84 for those
aged under 40 and 0*03 for those aged 40
or more.

(2) It is of interest to compare the
autumn 1978 results from the South West
Oncology Group's adult acute leukaemia
studies (personal communication from Dr
K. B. McCredie) with the MRC results in
our 6th AML chemotherapy and immuno-
therapy trials. Neither the British nor the
American studies have all cases of AML
referred to them, arid this "selection" of
patients might well exclude different
proportions of those likely to die most

rapidly from the two studies (for example,
a larger proportion of the British patients
were old). Nevertheless, the American
study is very large and it is noteworthy
that in every age-group there was a larger
proportion of remissions than in the
present trial. Overall, 54% of their 465
AML patients remitted, which is com-
parable with the 52% of 148 patients in
the MRC's AML6 immunotherapy trial
(MRC, 1978) and considerably better than
the 32% of 250 in the present trial. How-
ever, once remission was achieved, in the
SWOG studies the subsequent disease-free
survival was almost identical to that in
both the MRC AML6 trials. In particular,
a randomized comparison twice as large as
the MRC immunotherapy comparison

MEDICAL RESEARCH COUNCIL

showed no material difference between
OAP chemotherapy and OAP + BCG
chemoimmunotherapy. This is compatible
with the view (which underlay the recent

changes from the MRC 7th to the current
8th trial) that the overriding need is to put
the most intensive efforts possible into
AML remission induction.

82

CHEMOTHERAPY OF AML

o     eq        N     o       CO   CO
O     CO       eq    1        eq   to

0)    0)       0q    10       CO   CO
CO1  tN        CO    N       CO     NO

N     e>       CO    CO        4   CO
o     _         -    o        -     o

C3O   eq        C>   eq      eq    C
eq     CO             -       _

0)    ,O       10             o 0  C
eq    C         -_   -        eq   -

co CO 10 CO
'- .q CO -4

-4 '4 eq 10
- eq CO -

-0
eq O

cq V

CO
10

-

eo
Ci

tS

CO 0
~ V
S 141

'0 P1 (=>
ci> gn V

,0 ),    C O -  -  -

Nq O0)   -  N  CO Co
COCO0   10-  N  CO  CO

0       C  0   eq  CO
NN0)CO 4   t3   ND   0  0)>

t)e:~   V    A\0

6        eo

z

0~~~~~~~~~~0

z       V      A\SNwtz<X'

01

?  Cq  00 01 tX   O  t  0t O1
z        C     A

41eq0)   CO  Co  0 o CO >0
-   nC,0  'C)  0)C

?       . E9 H   H
E- ,? O  Od A =  f

eq  CO  -     a q  10
C;  q  CO  1 -q  CO

CO  N  10 C   N  C

CO C   ,  -0  -  0)
O -     - CO     O

CO  eq  1)  10 )

CO CO CO 10 -_
eq C   - -       -_

ce ok m rq
CO  eq N

e-

11 o
Im P   11

0)
eq

-

eq
eq

CO

in 11 .
0) 4l I

1 011

ot-

mm

COCO

1t 0N C   m 0l  Co  CDO  l4
t410t4CO  CO  10 Ct O  s4
oo 00- O - 0  - 0

0

74

E         -

V
PA

X

sCOC,-eq_ t

?4 N rCOCO i

C.)

H

o to

Vi 4

4  C   ~o

x

-

A

,  o  o  U. ,>

,q   E-- EN  pq   E
H    P.   HE4      .

'2 HX X

83

0
0

._

Q
4.4

0
C1)
4.')

0~

CO

tD-
fq

01

.)

Ca

-

Cs

W
;,  .
0

Ca

,tn

O%) 4 I

o

*4. CQ

.C.) 4

"Qo

4..'

tu  4 , X

C.)

o z

4!)

CO
E -

CO

tt X     0

CO CO
eq "

- .4

eq    CO

eq   C

CO CO
0 e

C> ,q
- IIO

CO 0
eq CO

CO 0)
- CO
-    0

0     0
CO 0)

0 10

eq -
-> _
_.   _i

cW z
0)

. '  E. t  [1

01

(D 0
t

>   0o

Ca)
on d 0

O

m

O 01

D~
C)

>

00

01.

fri -4. 14 E

a ) ,O   01 C

>

.C)

0~
0
;I4

10
eq

?O
H-

z
E-4

E.1
41

aq

P.4
?

MEDICAL RESEARCH COUNCIL

e(
- o

i 6NII

CJ  10.

0,

0,
14

40

0       3

4a00       0

"0

0

0,

14.4 0

0
44

-0
<x         aq 11

t:. PL 1

r-0             m

1-Z       xZ         m

N   00   CO   00   o    0        C
-   10   CO   CO             -   C

Ct  10   00   10   o    o        00
CO  N     N   N    CO   0    N   m

o   00    0   0     N   CO   C   CO
(N   O    0.  0     0   0    0.   0
C         o Ao  -       -     _o

b         cc t      NN        - so  or

CC   0    -   o
CO  N  0  N  N  00   (N~~~~~~~~r-  0-

0

P-

co
CO
(N4

G0      I"      0 Ox

AZ      1~Z X    Z~

10   10       (N   0      00    0      (N    co
(N    =      CO    CO      -    (N     cq    -

00    00
010        00

0   1-4    oo

V

D    h    ts
0    -

CO            N
0       V

A "s

?     % cq        t-

0         P-4     P--(
pq   44

1;t  A    ?-4

-o-Z  "
9

1-4  rp   M

E--4   114

N    'Z?  I       --!?
m

4                P4

E-4       PA      E--l

0

V

"-

40' I
X
0
4-

A\

o)  I
I

%)I

00 NO

A\

N COOo.

000 t

-  0'-

X

0-

V

10  00~

(N

-    X
c-_

.-

00 _ -1

EH

IM?

CO  tt

A\

ot b

to C r

P-

A\

COCO

0 0 0

0

NO COO0
m4 004 0
.' CO,H

M        M
E-  X

10

O
0-:

-1
la

11*0  coj

C* m z  m

00    -q        00   N-      CO     0
w     N         10    N-      N     co

0     o         0     o       I    0
00   (N        (N4   00o      -    00

(6              P- 4  C>      1-    O
4     (N -;           (N      -     P-

00    00        0>   00       '4 t

CO    (N       (N    -        -4    1-

Co " C   00           CO      10    Co
(a              (N _ _  es  c(  -   -

10

-0

I1

0,
- -

00oo cOO = 4 00  C o
eC c  N c 4  C  (N  c(

z

E. 410 "CO

z

= 0 1   00
m 00__co

rA 6 61AA -:

p4

-4

0

x

0

0

co

E4
A

*.4

E-1

tN            (N     -
04    10      CO    a4

P-    00      P-    0M
-     0        -     0

N-CO    Q.  10~  CO  .   1  co

0400 (        0 4t- x to t

10 0otot

x         x

1t0  Nc _  C         O   1  G0

010 cc  t  t  wa  e  cc -4a

V   ciA\O        E-i     H C

84

0

0

.4

m
C4.4
0

.>

-4a

"0*

0

-44

0,

0

._-

P4

to

0

00

L

.0
0 .

* 0

0-4

0

14
0

CHEMOTHERAPY OF AML

1 t  t to

t- Nq t  0 QC

C>   rr  oo-4 <=

000o C C  00  o
cO r-S  s

N  01  N0  00

_ oC0  _O00o  _ o

C)3 N   N 0

-O -O O O

0   0 o- c   o to

--  6  A 6 P--  -

0 0 0   _ 1  0 0 _

000   000   o o o

_40 _ 0_  _ O _
_ _  _ _  _ _-

ce X cs _ ce  0
c Z  A Z  A ZX  e

0 1 0   _ _N   N 1 0

0 1  0 1 0 e

C  O~   O CO_ _

b4't  00'. t   C

0 0 1 c0   0 1 00b

- 0  c0   0 1 00s

0    0 cDD   c

0 0   1 0 1   0 0t  c
No   es   1 o 000

*. ~  . ..

o   00
~ ?

_ ca  u o  \ E

-o
_11

MN 10

00  o
b4 -

0 -

CO 0
oo

P4  01

N   CO
r 0
CO 01

&
01  10

00 10
t4 0

z

oN 01C

S? 0 -
z

ri3

X- _  0 n

@3M *
- - 00

S 10N*

@3X

@3o b

0

EzC

m

..0* S

0 _

-   .n D

> ?;

03

co
1:

03

03

CC~

CC4

CC4
03

C

IC

03

C

IC

Cim      aSF tot

~00      000to

aq        00 ce

01

0 aD        aD

I         *4  0

I   00      00

00 C       COaD

0 0     *   C

0    O       C O

0      __

^ 10      _14

00 -5       s a

10 aD         N
UI  0 1    -

0           1

q   4 aD   D C

00 * N

00 -_

ii_ b00

0 0100_
I    00      01

00

t-   _ t

g H-

.tD 22

?   M

CA)m

?   0  C 0

z
0

E4
z

v

@3

m

@3

0

0

E-4

0

"I

110    00  01  0014  101o
CO  0   0O   00  0   0 0

OCOWn  c  ~aq  sc   3

- - 0   -   = A ~

- 0 0   01  00  100N  0 -
0000   0   0 1 0 C O

-q a   01   4  01  CO 0 0
__ OC _00  0  N  O N  1

int t  c  - a100  0000
C O - 4 1 1 0   C O 0   1 0   0 0

0 0 0 0 a-   0 0  01 0 0o4

)01

1 N 1  CO  0  10  000

- -0 0   0  -  O   0  -

C O10CO 4  10  ND U   t   Da
-   o

@3

0 4 0 0 0   11 0  0Z  0 s 1 0 0  Cs

- >; CO10C  O 4  CO  NO O C

s C0 O ff a

Ci   _  b  $0 O   5  A s

__oo   po     _ _

aD i  aD    CS a

U: l At  e  b O    --~

85

-4o

10

*O4110

00 00
010010

G aq
0 O0

_q m es

000

aq q <
00000
C1 01

u 00 0

01
ED ~-COC

x 010100

-V

Zr 110
0   0

o 00010r

t 001 _ O

zS

0

c) 1

0 oc

c)Xs  4t

86                         MEDICAL RESEARCH COUNCIL

TABLE IX.-Effects of retrospective stratification for various presentation features on the

difference in overall survival associated with treatment

Features for

which treatment

comparison is

adjusted
None
Age

WBC

WBC and Age
Blasts
Hb

Liver palpability
Centre

BARTS III

Observed      Extent of

no. of    exposure to risk
deaths       of death*

(0)            (E)

120            106-0
120            105-2
115            103-7
115            104-5
115            101*8
114             99*8
115            106-7
120            108-2

TRAP

0            E*

115          129*0
115          129*8
114          125*3
114          124-5
114          127-2
114          128-2
114          122-3
115          126-8

* Retrospectively stratified for one feature (see Peto et al., (1977) section 22). The observed numbers vary
slightly because patients for whom a particular feature was inadvertently not recorded at presentation are
excluded from the corresponding adjusted analysis of survival.

x 2

3*41
3*92
2*36
2-03
3-19
3*66
1-31
2*51

				


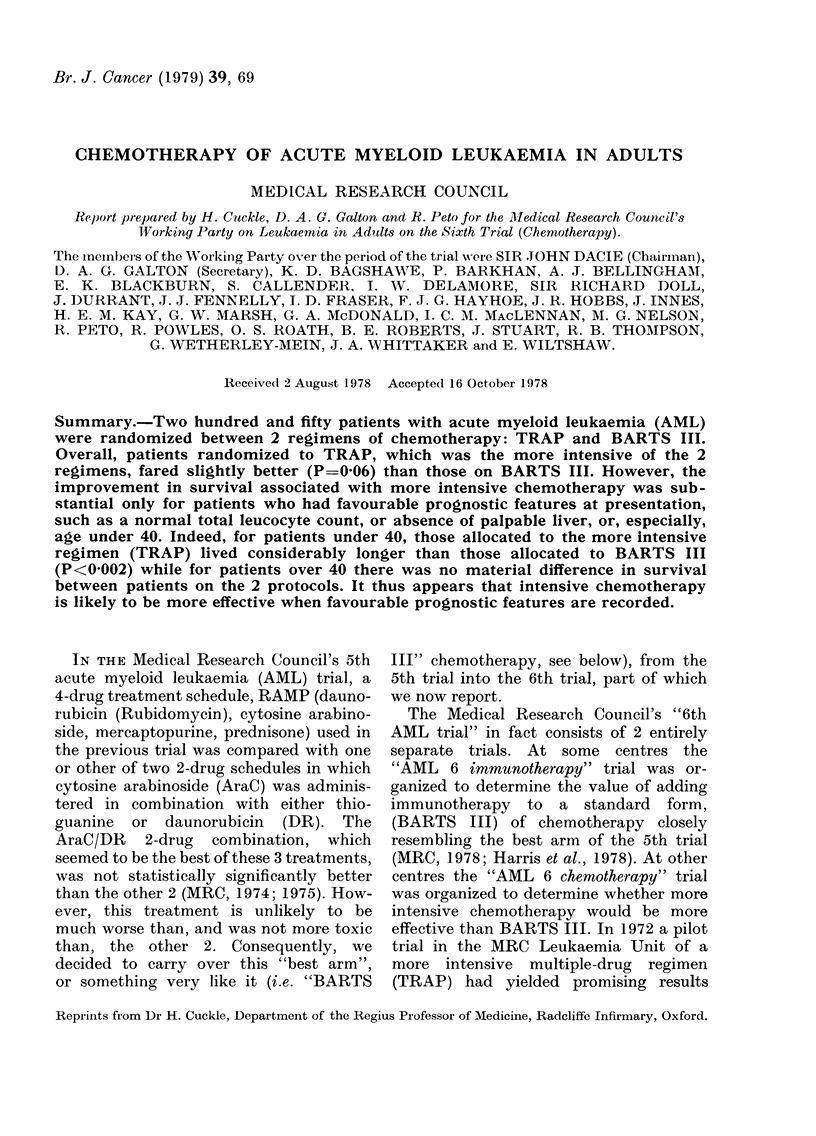

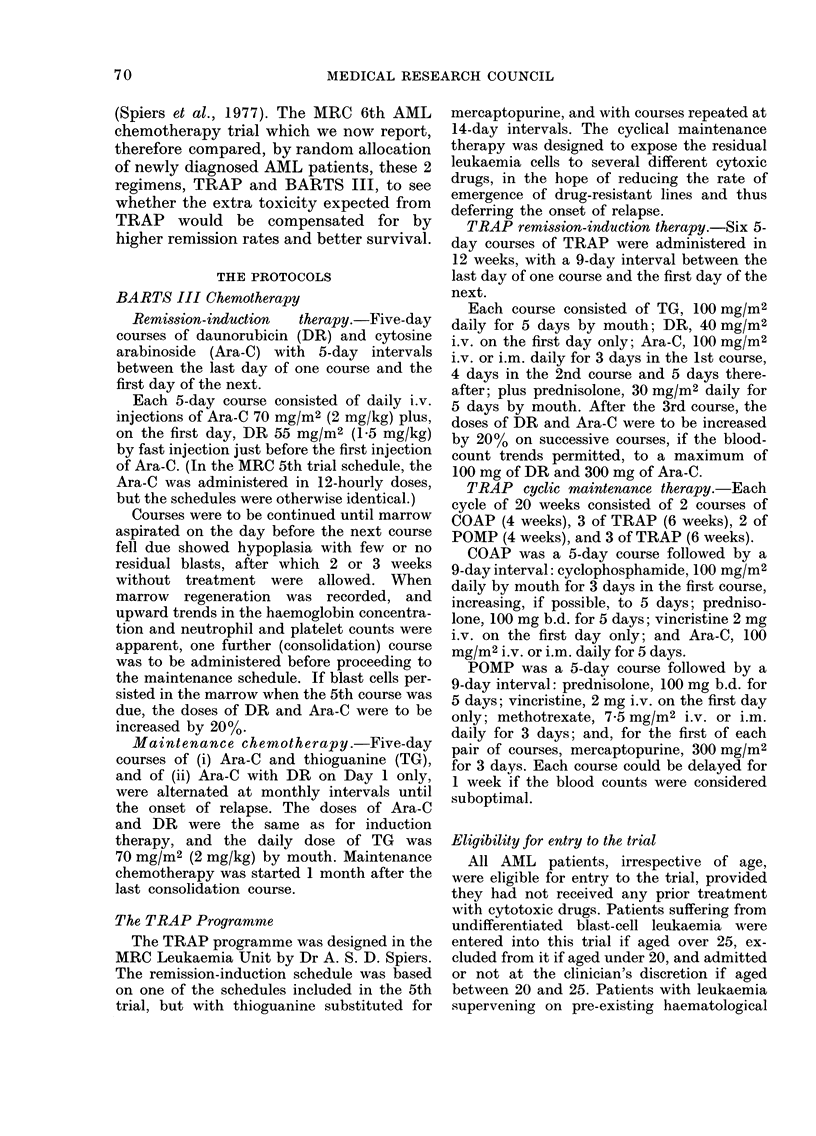

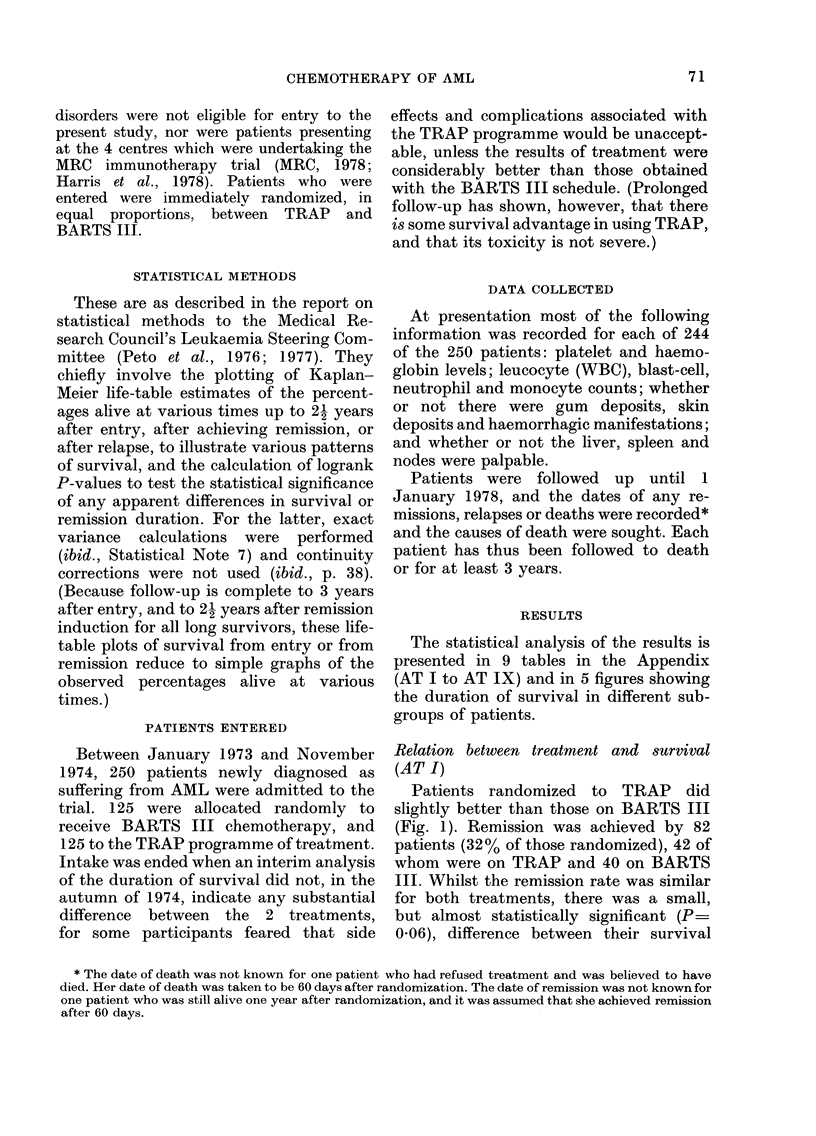

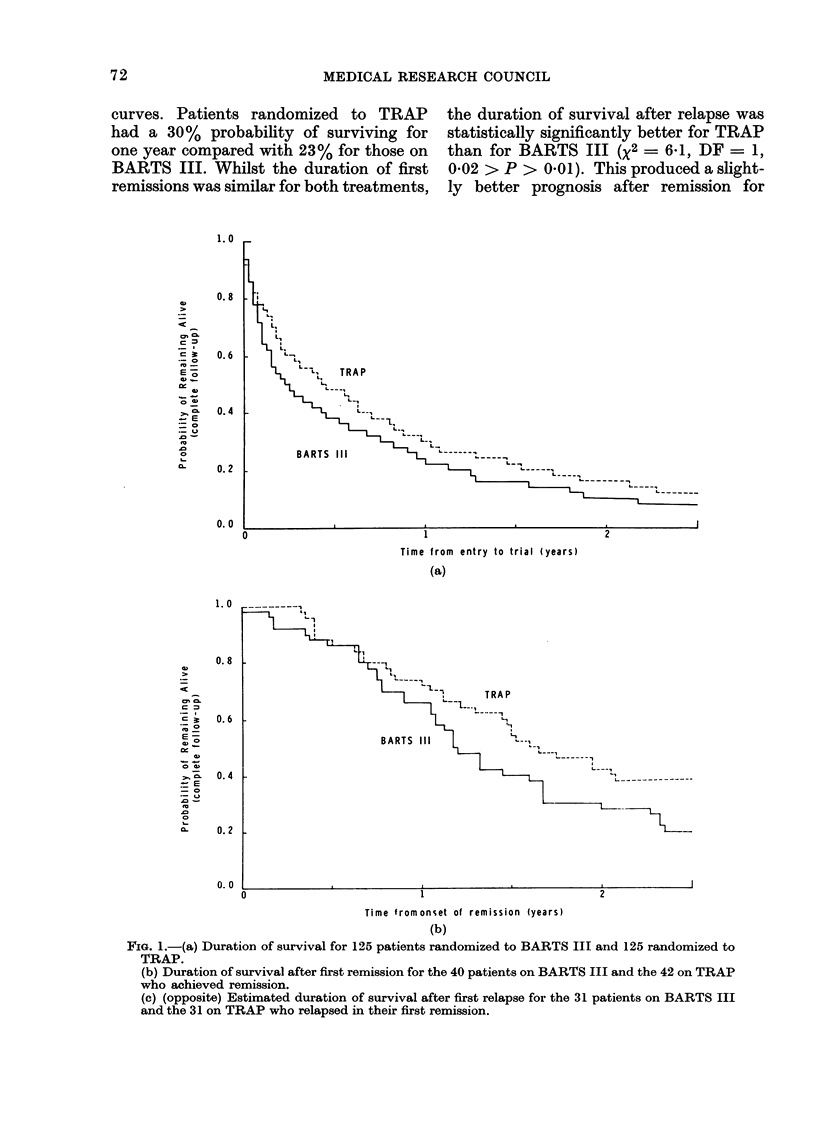

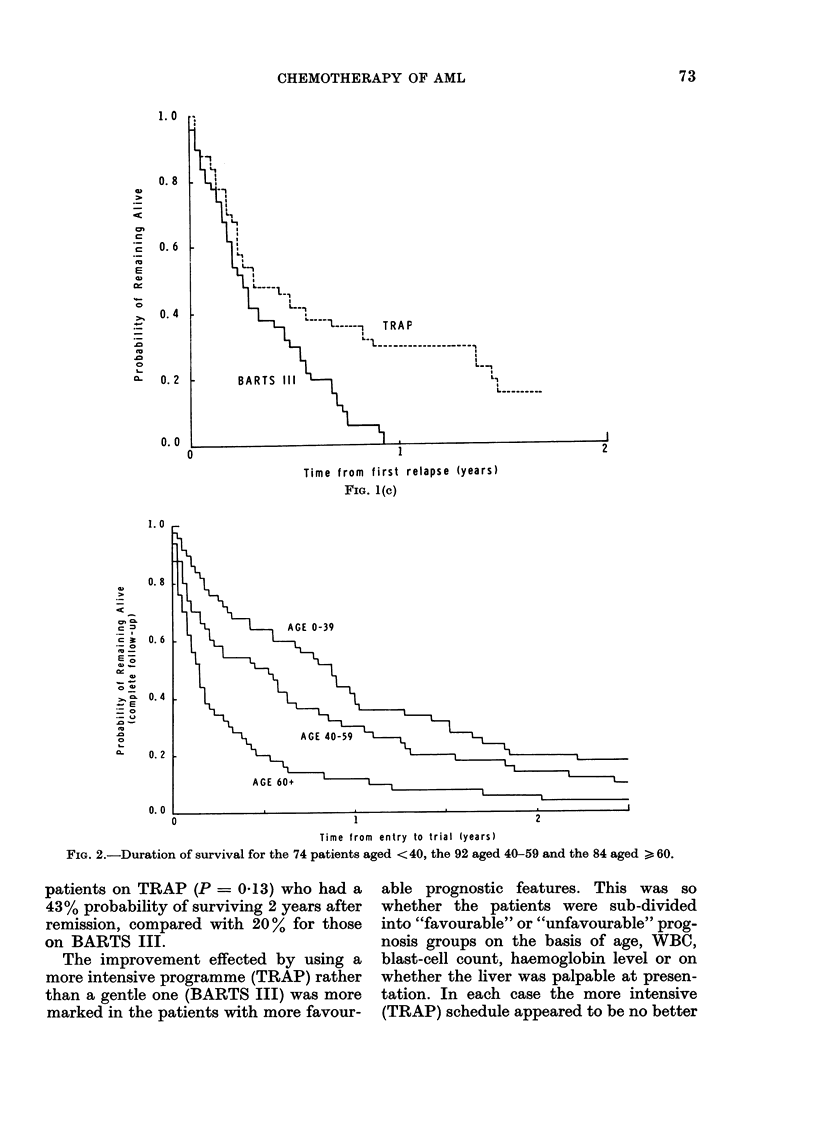

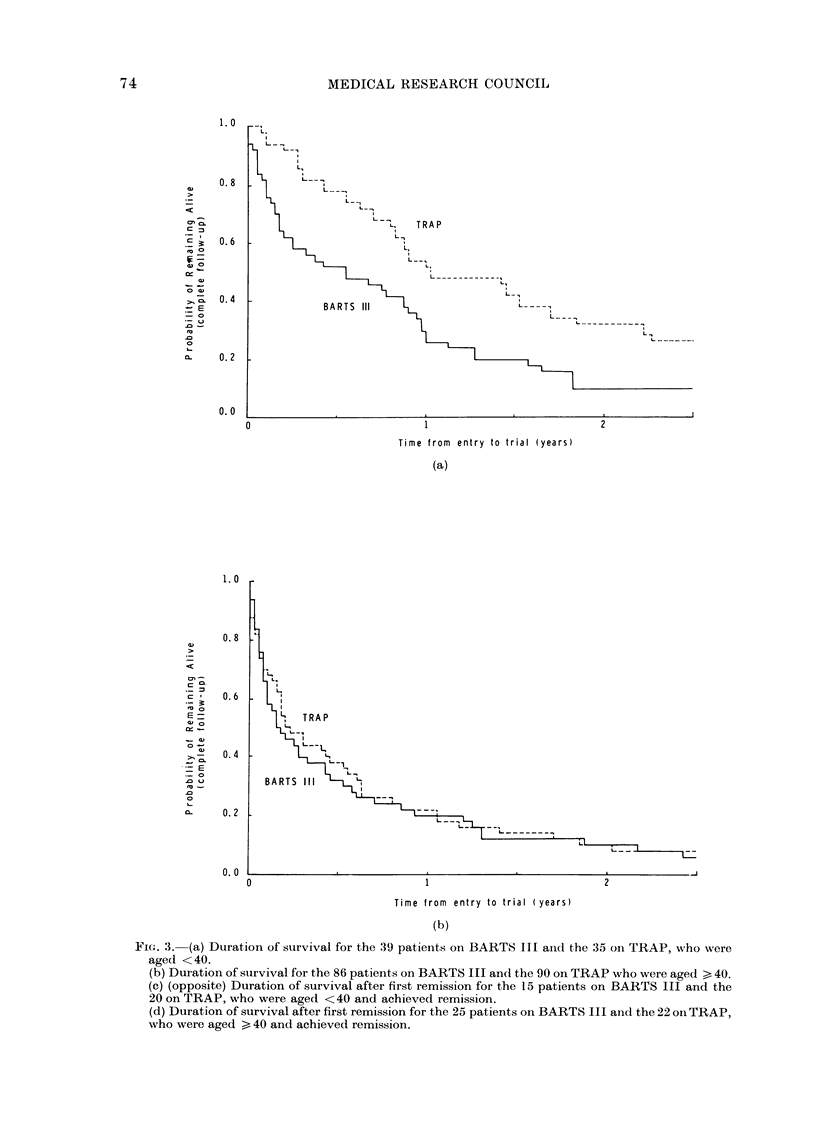

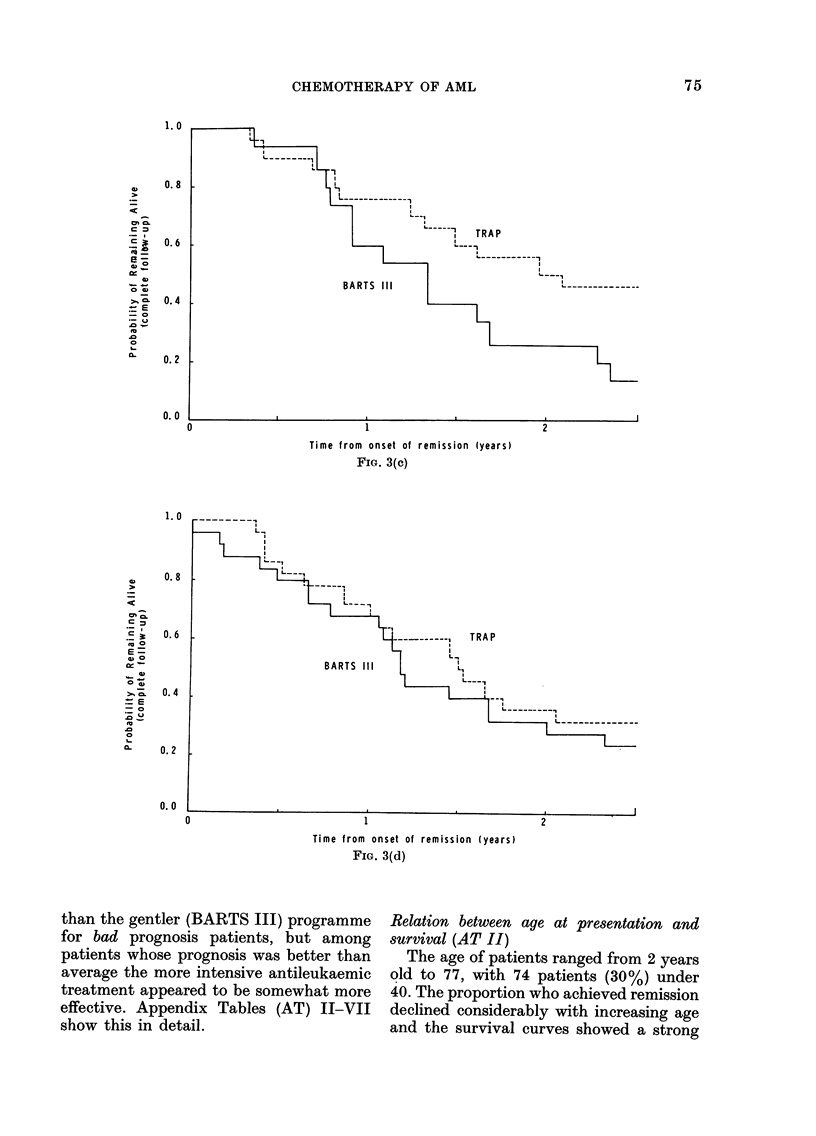

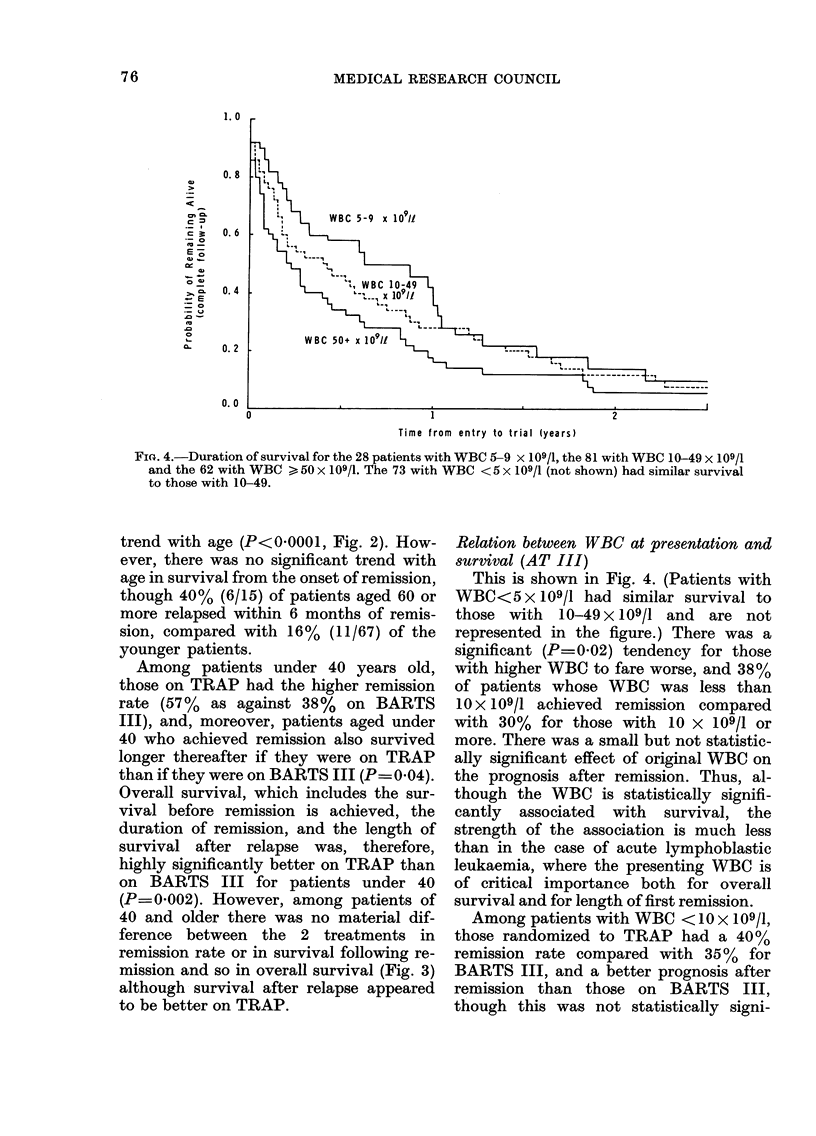

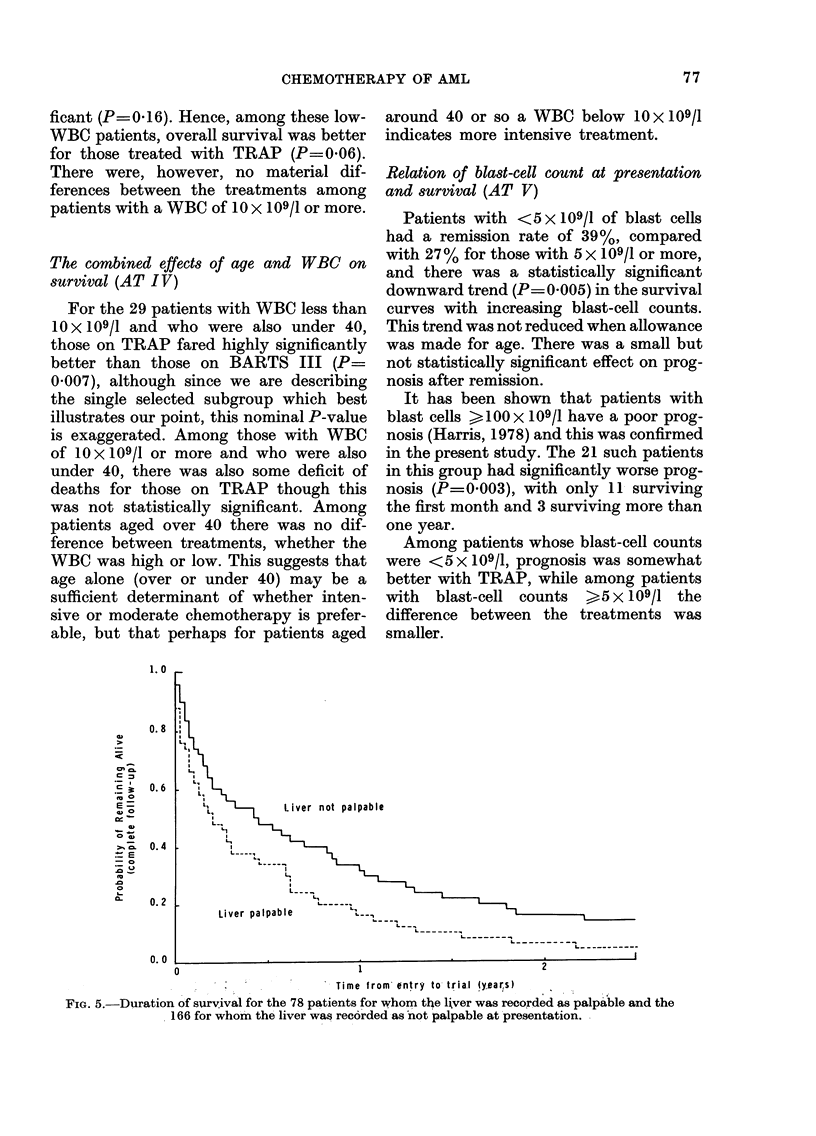

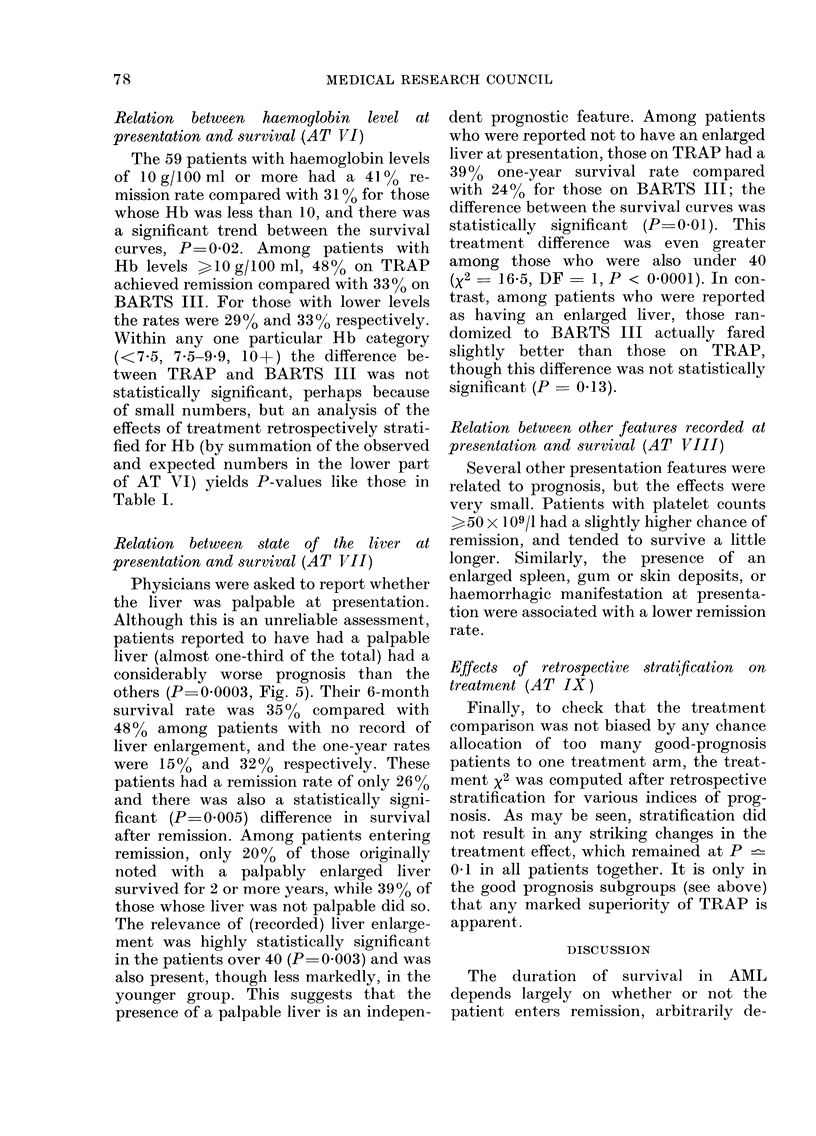

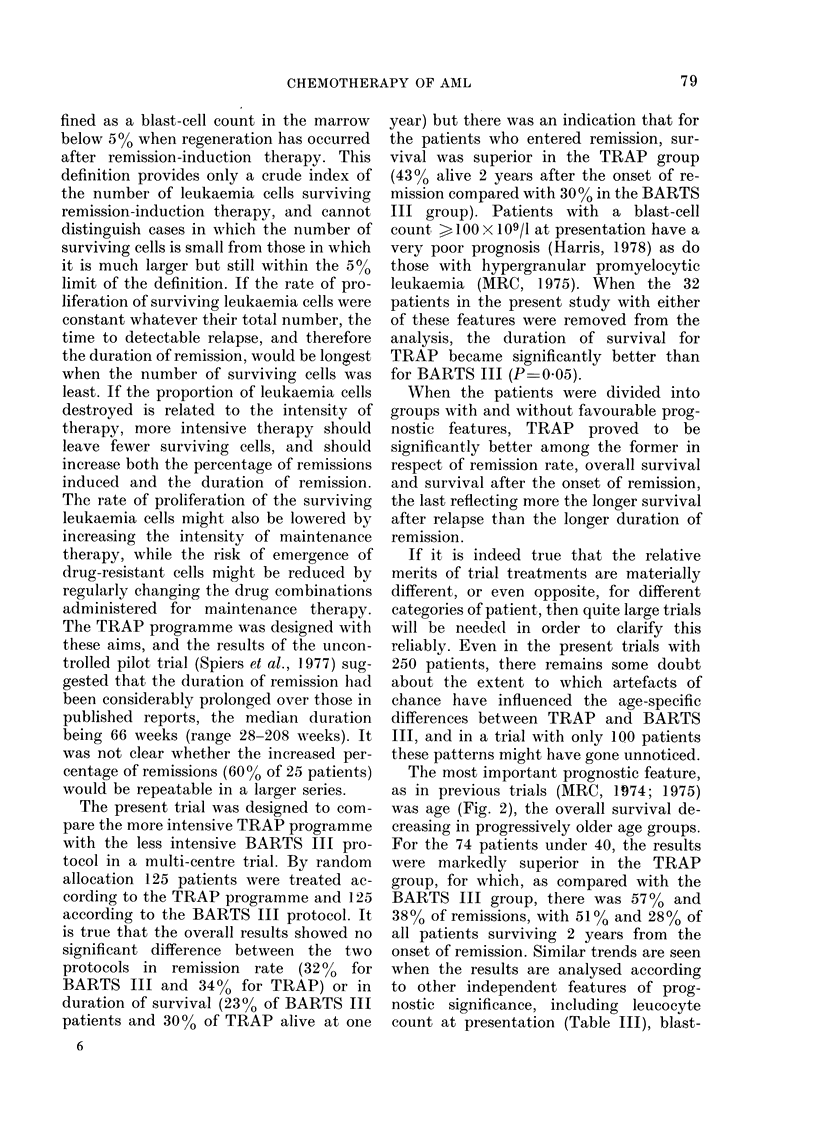

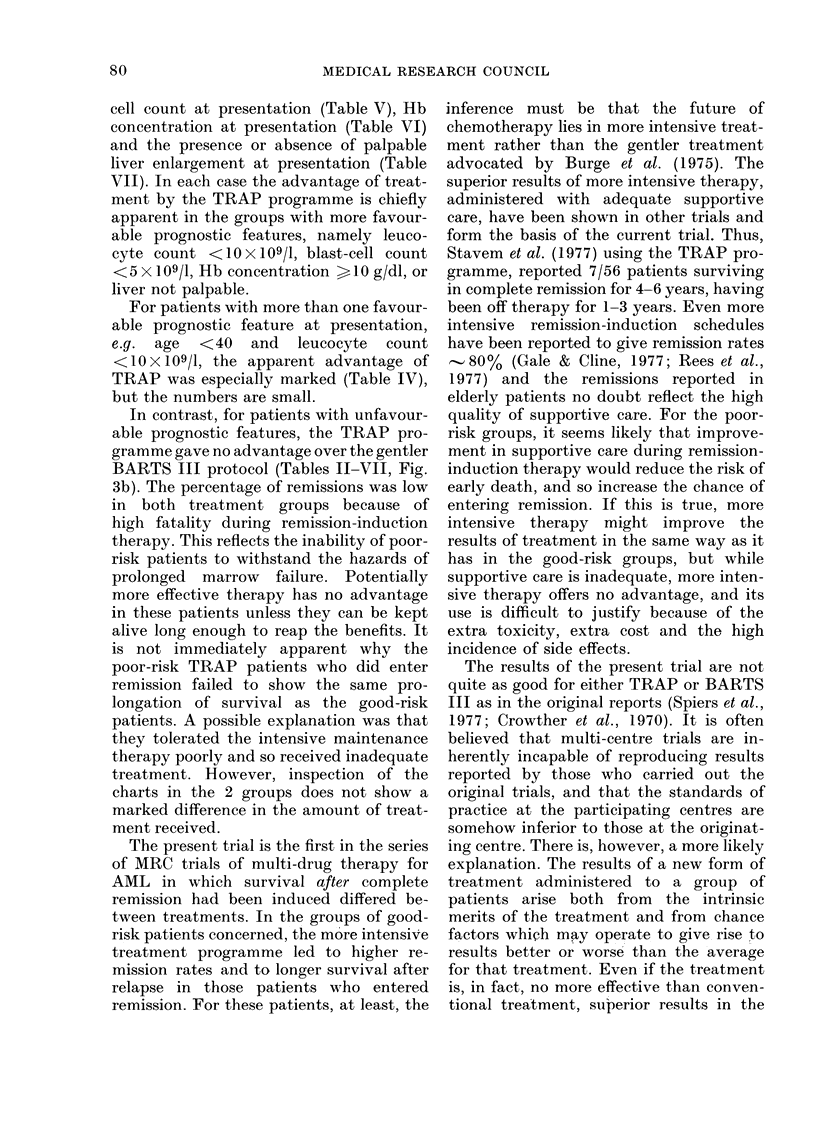

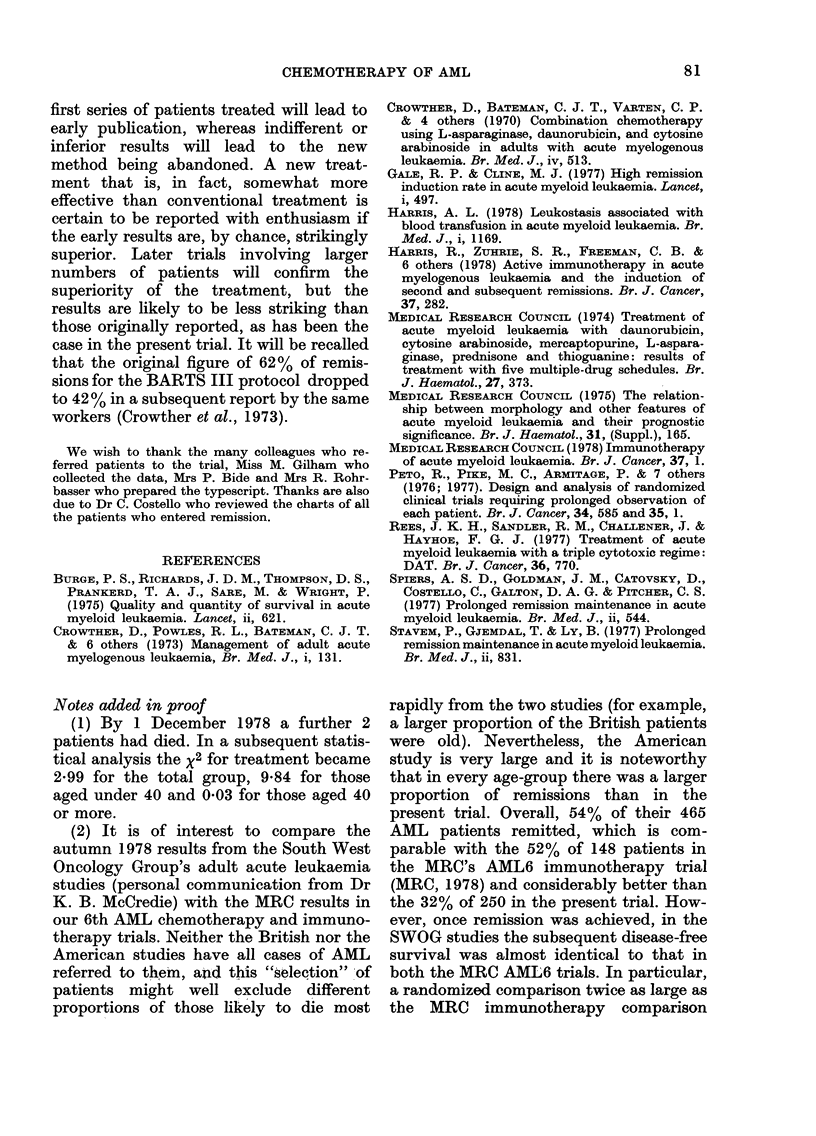

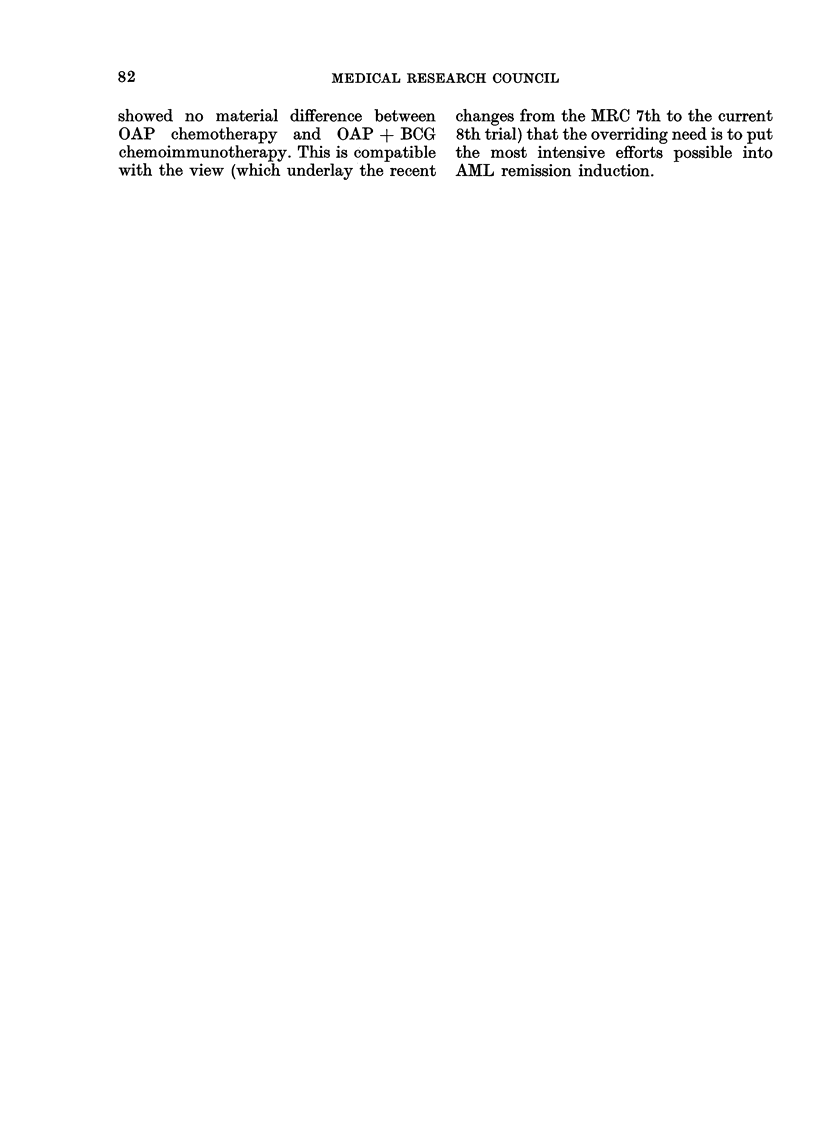

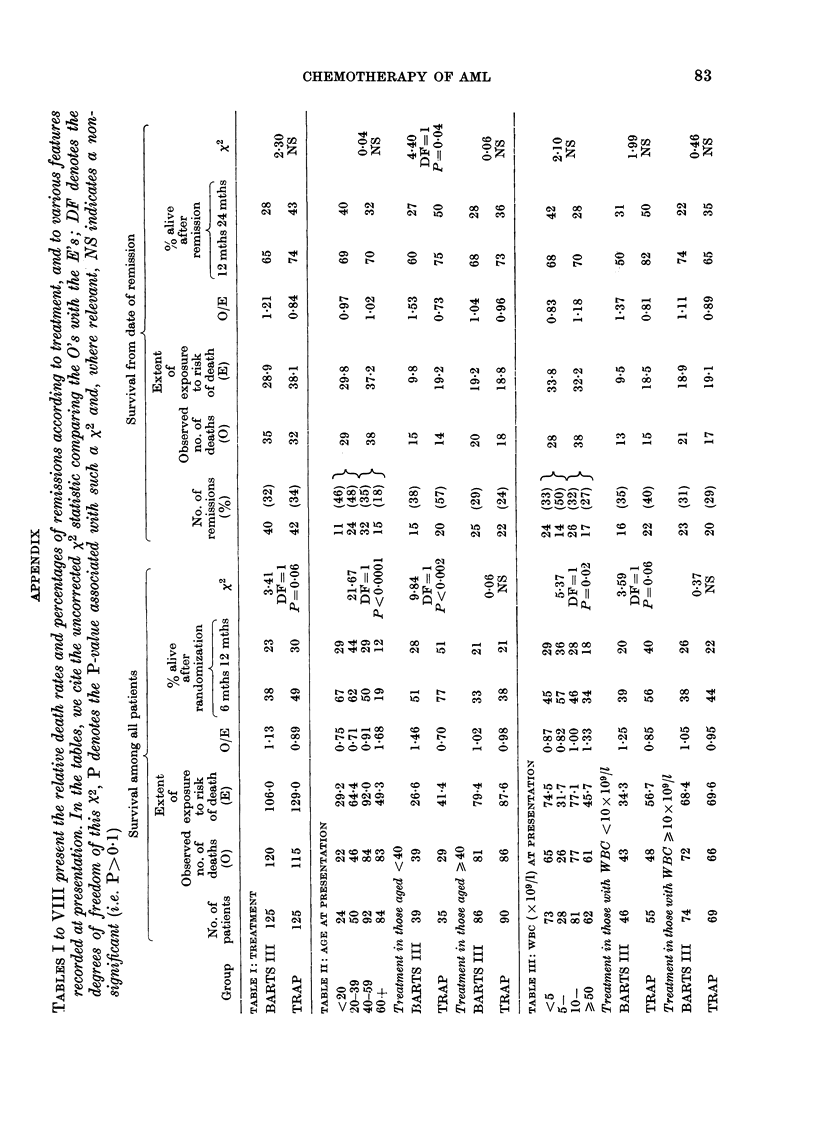

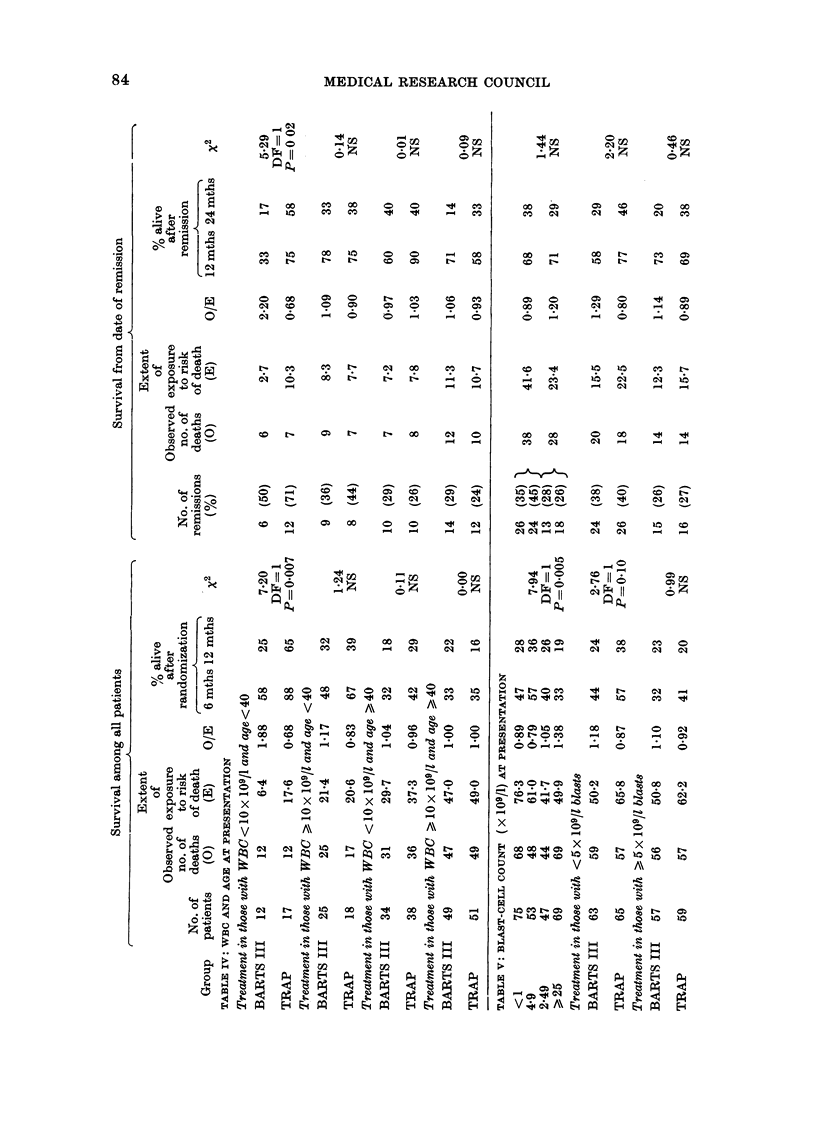

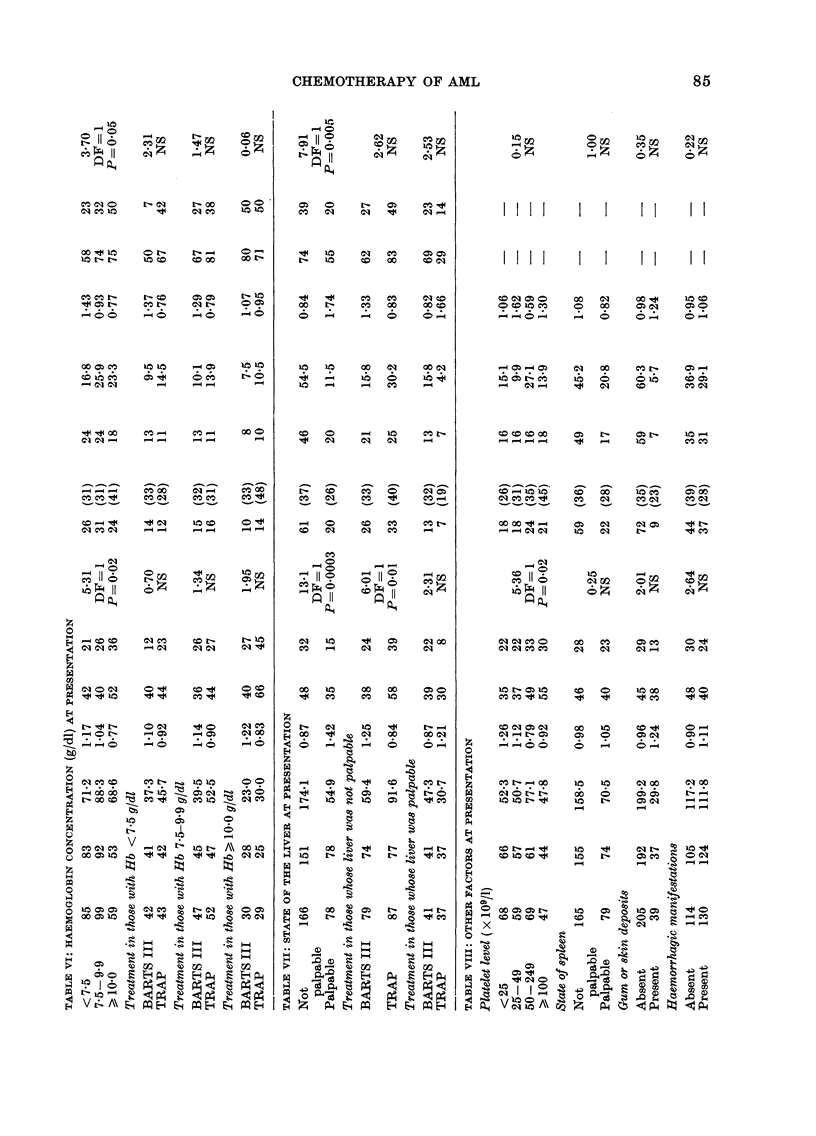

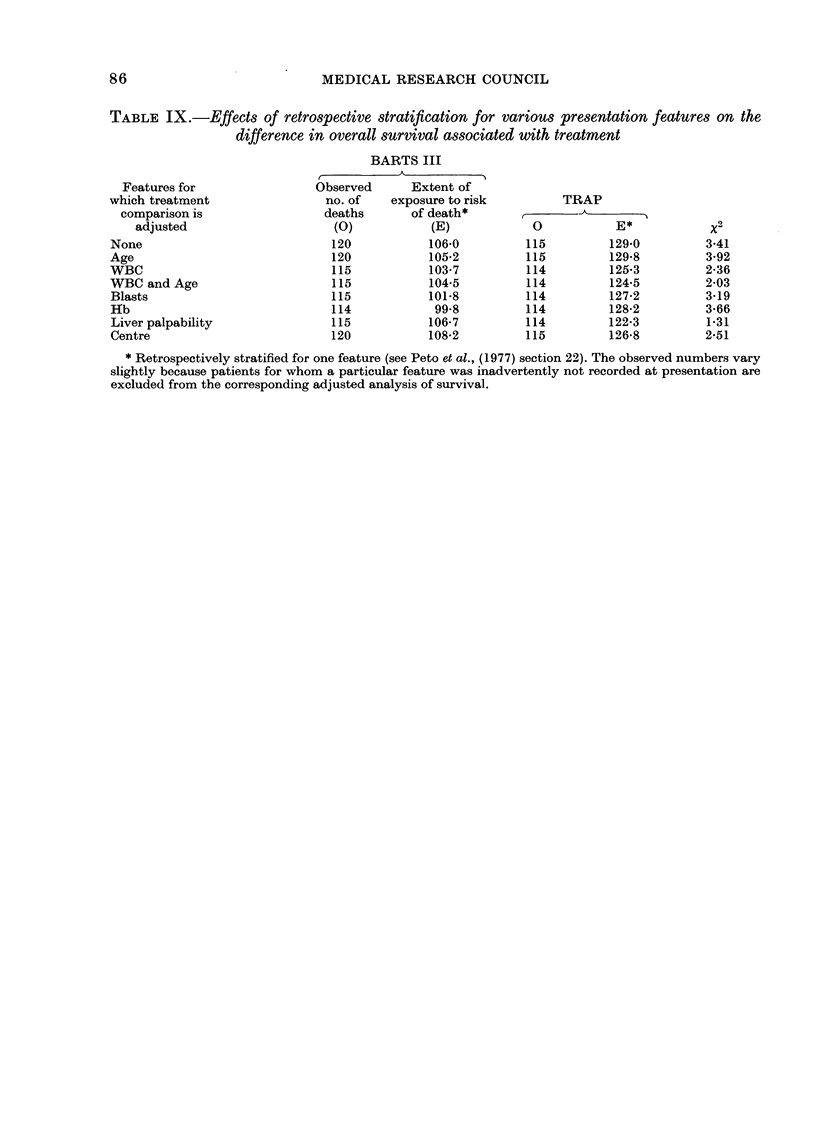

